# How to differentiate the fetal velamentous vein from maternal blood flow in cases with vasa previa

**DOI:** 10.1002/pd.5771

**Published:** 2020-08-20

**Authors:** Kensaku Nakai, Daisuke Tachibana, Mie Tahara, Takuya Misugi, Masayasu Koyama

**Affiliations:** ^1^ Department of Obstetrics and Gynecology Osaka City University Graduate School of Medicine Osaka Japan

**What's already known about this topic?**

The identification of vasa previa still remains difficult even for well‐trained obstetricians. Distinction of fetal from maternal veins is especially challenging because both have a slow blood velocity.

**What does this study add?**

Changes in venous flow with maternal respiration in vessels around the uterine cervix or lower uterine segment differentiate maternal from fetal vessel.

Vasa previa (VP) is a serious obstetric complication that can result in fetal hemorrhage due to the laceration of unprotected fetal vessels, which run though the membranes near the internal cervical os.^1^ Although transvaginal color Doppler ultrasonography is a highly useful tool for the detection of VP, the identification still remains difficult even for well‐trained obstetricians. This is especially the case when it comes to branches of umbilical veins, which have a slow blood velocity similar to that of maternal vessels perfusing the periphery of the placenta or veins in the lower uterine segment. Herein we describe a useful technique to differentiate fetal from maternal vessels in cases of suspected VP.

## CASE 1

**FIGURE 1:**
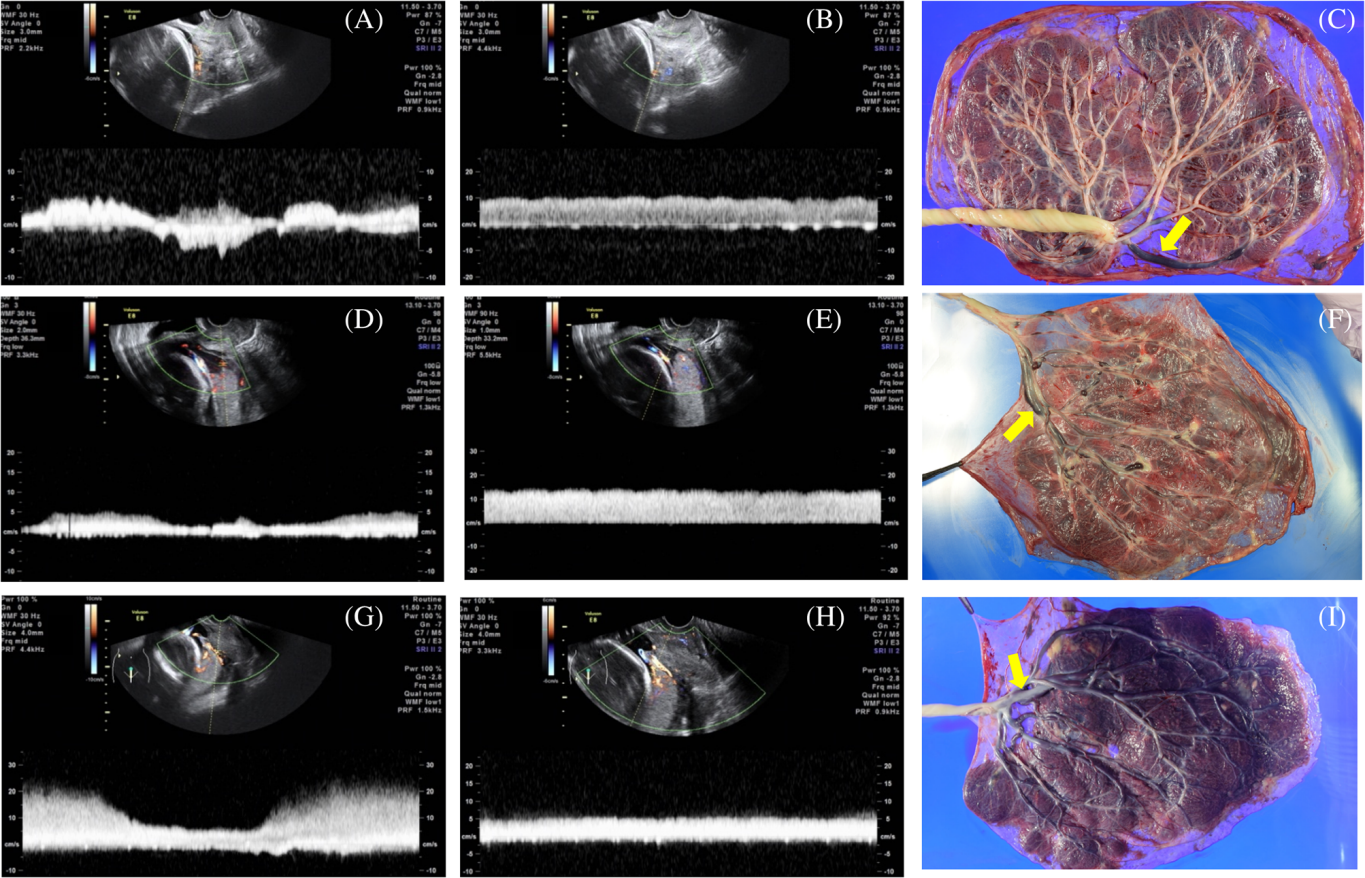
A‐C, D‐F, and G‐I show the trans‐vaginal ultrasound images and placental findings of case 1, case 2, and case 3, respectively. Fluctuation of venous flows were observed simultaneously with maternal breathing movement (A, D, G), although constant flows without maternal breathing movement were found in the fetal veins (B, E, H, respectively). In all cases, velamentous insertion of fetal veins was confirmed (Arrows in C, F, I, respectively)

At the 29th gestational week (GW), a transvaginal ultrasound revealed venous flow in vessels near the internal cervical os which was difficult to identify whether they originated from the fetus or the mother. At color Doppler ultrasound, two types of venous flow were detected: one fluctuated simultaneously with maternal deep breathing (Figure 1A), and the other showed a constant flow (Figure 1B). Based on these findings, we assumed that the vein with the fluctuations was maternally originated, whilst the other was a fetal vein running transversely near the uterine cervix. Fetal vein bridging the bilobed placenta was confirmed after caesarean section (Figure 1C, arrow). Case 2 and case 3: a transvaginal ultrasound revealed low‐lying placentas and adjacent vessels with venous flow (case 2; 24th GW: case 3; 23rd GW). Venous flow near the uterine wall showed fluctuations simultaneously with maternal breathing (Figure 1D,G), whereas the vessels near the placentas showed a constant flow (Figure 1E,H). We assumed that the vessels with constant flows, which were not disturbed by maternal breathing, were fetal veins. In both cases, fetal veins near the placental parenchyma were observed after a caesarean section (Figure 1F,I for case 2 and case 3, respectively).

In order to identify VP in the presence of abnormal cord insertion (eg, velamentous umbilical cord), obstetricians should pay careful attention at adjusting machine settings (ultrasound frequency, gain, scale, dynamic range, etc.), not only because the blood flows at a very low speed in fetal veins, but also because fetal vessels may run transversely near the internal cervical os as shown in our case 1, which might be to overlooked from the sagittal and parasagittal views commonly taken at transvaginal scans. Furthermore, retroplacental or periplacental maternal blood flow near the cervix should be distinguished from VP, since abnormal placentation (eg, migrated placenta previa or bilobed placentas) is highly associated with VP.^2^ We suggest that the presence of flow changes associated with maternal respiratory movements points to a maternal origin of a vessel. Our recommendation stems from a postpartum observation that blood flow in the uterine vein was reported to be affected by inspiration movement.^3^ We cannot comment as to whether also vessels located in other areas, for example, at uterine fundus, may be affected by maternal diaphragmatic movements, but it seems that venous flow around the uterine cervix reflects maternal breathing motion. Our findings will provide clinically important clues for the identification of fetal vessels around the internal cervical os, whenever VP is suspected in the presence of abnormal placental or cord implantation.

## CONFLICT OF INTEREST

The authors declare no potential conflict of interest.

## DATA AVAILABILITY STATEMENT

No research data.
